# Optical Coherence Tomography and Optical Coherence Tomography Angiography Findings After Optic Neuritis in Multiple Sclerosis

**DOI:** 10.3389/fneur.2020.618879

**Published:** 2020-12-15

**Authors:** Olwen C. Murphy, Grigorios Kalaitzidis, Eleni Vasileiou, Angeliki G. Filippatou, Jeffrey Lambe, Henrik Ehrhardt, Nicole Pellegrini, Elias S. Sotirchos, Nicholas J. Luciano, Yihao Liu, Kathryn C. Fitzgerald, Jerry L. Prince, Peter A. Calabresi, Shiv Saidha

**Affiliations:** ^1^Division of Neuroimmunology and Neurological Infections, Department of Neurology, Johns Hopkins Hospital, Baltimore, MD, United States; ^2^Department of Electrical and Computer Engineering, Johns Hopkins University, Baltimore, MD, United States

**Keywords:** outcomes, inter-eye asymmetry, retinal vasculature, optical coherence tomography angiography, optical coherence tomography, optic neuritis, multiple sclerosis

## Abstract

**Background:** In people with multiple sclerosis (MS), optic neuritis (ON) results in inner retinal layer thinning, and reduced density of the retinal microvasculature.

**Objective:** To compare inter-eye differences (IEDs) in macular optical coherence tomography (OCT) and OCT angiography (OCTA) measures in MS patients with a history of unilateral ON (MS ON) vs. MS patients with no history of ON (MS non-ON), and to assess how these measures correlate with visual function outcomes after ON.

**Methods:** In this cross-sectional study, people with MS underwent OCT and OCTA. Superficial vascular plexus (SVP) density of each eye was quantified using a deep neural network. IEDs were calculated with respect to the ON eye in MS ON patients, and with respect to the right eye in MS non-ON patients. Statistical analyses used mixed-effect regression models accounting for intra-subject correlations.

**Results:** We included 43 MS ON patients (with 92 discrete OCT/OCTA visits) and 14 MS non-ON patients (with 24 OCT/OCTA visits). Across the cohorts, mean IED in SVP density was −2.69% (SD 3.23) in MS ON patients, as compared to 0.17% (SD 2.39) in MS non-ON patients (*p* = 0.002). When the MS ON patients were further stratified according to time from ON and compared to MS non-ON patients with multiple cross-sectional analyses, we identified that IED in SVP density was significantly increased in MS ON patients at 1–3 years (*p* = < 0.001) and >3 years post-ON (*p* < 0.001), but not at <3 months (*p* = 0.21) or 3–12 months post-ON (*p* = 0.07), while IED in ganglion cell + inner plexiform layer (GCIPL) thickness was significantly increased in MS ON patients at all time points post-ON (*p* ≦ 0.01 for all). IED in SVP density and IED in GCIPL thickness demonstrated significant relationships with IEDs in 100% contrast, 2.5% contrast, and 1.25% contrast letter acuity in MS ON patients (*p* < 0.001 for all).

**Conclusions:** Our findings suggest that increased IED in SVP density can be detected after ON in MS using OCTA, and detectable changes in SVP density after ON may occur after changes in GCIPL thickness. IED in SVP density and IED in GCIPL thickness correlate well with visual function outcomes in MS ON patients.

## Introduction

Retinal atrophy detected using optical coherence tomography (OCT) has emerged as a promising biomarker of neurodegeneration in multiple sclerosis (MS), demonstrating strong structure-function relationships, correlations with global measures of disease activity and disability progression, and differential modulation by disease-modifying therapies (1, 2). Retinal optical coherence tomography angiography (OCTA) represents a more recent evolution of OCT technology, offering a rapid, non-invasive, and inexpensive technique for examination of the retinal vasculature (3). The ability to image microvascular structure and therefore potentially metabolic demand of retinal tissue offers unique avenues for investigation in MS—a disease in which the anterior visual pathway is a key site of injury, and in which metabolic changes may play a crucial role in the pathway from inflammatory tissue injury to neurodegeneration (4, 5).

Studies employing OCTA have shown that retinal vascular plexus densities are reduced in MS—particularly within the superficial vascular plexus (SVP; which mainly supplies the ganglion cell layer), and the greatest reductions in SVP density are detectable in eyes with a history of optic neuritis (ON) (6–9). Relationships between OCTA measures and disability [both expanded disability status scale (EDSS) score and visual function] have been suggested in people with MS (6, 8). Furthermore, in one study, SVP density demonstrated significant relationships with multiple sclerosis functional composite (MSFC) score, while GCIPL thickness and MSFC scores were unrelated, suggesting that SVP density may provide additional information beyond retinal layer thicknesses as a biomarker of functional disability in MS (6). It is not yet known whether (1) increased IED in SVP density can be detected in individuals with MS after ON, (2) whether IEDs in SVP density may correlate with visual function outcomes after ON, and (3) how these relationships may compare to IEDs in retinal layer thickness measures after ON.

In this study, we aimed to establish whether IEDs in SVP density are greater in MS ON patients than in MS non-ON patients. Additionally, we set out to compare IEDs in OCT and OCTA measures after ON, and explore the relationships of these measures with visual function outcomes.

## Materials and Methods

### Study Design and Participants

People with MS were recruited by convenience sampling from the Johns Hopkins MS Center for this cross-sectional study. Diagnoses were made according to the 2017 revised McDonald Criteria (10). We included patients with high-risk clinically isolated syndrome (CIS) or relapsing remitting MS (RRMS), and excluded patients with primary progressive MS (PPMS), secondary progressive MS (SPMS), seropositivity for myelin oligodendrocyte glycoprotein IgG (MOG-IgG), or seropositivity for aquaporin-4 IgG (AQP4-IgG). Patients were stratified into two groups according to ON history; (1) patients with a history of a single episode of unilateral ON [MS ON patients], and (2) patients with no history of ON in either eye [MS non-ON patients]. History of ON was established from the patients' medical records, with all diagnoses of ON made by attending neurologists with extensive clinical expertise in neuroimmunology. We excluded patients in whom (1) the diagnosis of ON was uncertain, (2) any elements of the history or ophthalmologic examination were suggestive of an alternative diagnosis (e.g., ischemic optic neuropathy, retinal artery, or vein occlusion), (3) the laterality (i.e., right vs. left) of an ON episode was unclear, (4) the history included multiple episodes of ON, or (5) the history included bilateral ON. Additional exclusion criteria included relevant known neurological or ophthalmological co-morbidities (e.g., glaucoma, macular degeneration, history of any other relevant retinal pathology such as retinal vascular occlusion or retinal detachment), prior eye trauma or ocular surgery, refractive errors of >6 or < -6 diopters, poorly-controlled hypertension, or poorly-controlled diabetes mellitus. All patients in the current study received routine, high-quality ophthalmological care. This is a standard of care for all patients with MS receiving care at our center, and therefore makes confounding from any other ophthalmological disorder highly unlikely.

### OCT and OCTA Acquisition, Processing, and Quantification

OCT and OCTA scans were acquired under low-lighting conditions without pupillary dilation by experienced technicians, as described in detail elsewhere (6, 11, 12). OCT scans of the optic disc and macula were acquired using Cirrus HD-OCT (model 5000, software version 8.1, Carl Zeiss Meditec, California, United States), as our standard research protocol during the study period was to track retinal layer thicknesses in all patients using Cirrus HC-OCT, followed by Spectralis SD-OCTA imaging, and only to complete additional Spectralis SD-OCT imaging of retinal layer thicknesses where it was not burdensome on the patient. Acquired images underwent quality control in accordance with the OSCAR-IB criteria (13). Peripapillary retinal nerve fiber layer (pRNFL) thickness was quantified by the conventionally incorporated Cirrus HD-OCT algorithm. Macular retinal layer thicknesses (ganglion cell + inner plexiform layer, GCIPL; inner nuclear layer, INL; outer nuclear layer, ONL; average macular thickness, AMT) were quantified using an algorithm developed at Johns Hopkins University that has been extensively validated and utilized in both cross-sectional and longitudinal studies across multiple OCT devices (12, 14–16)

**Figure 1:**
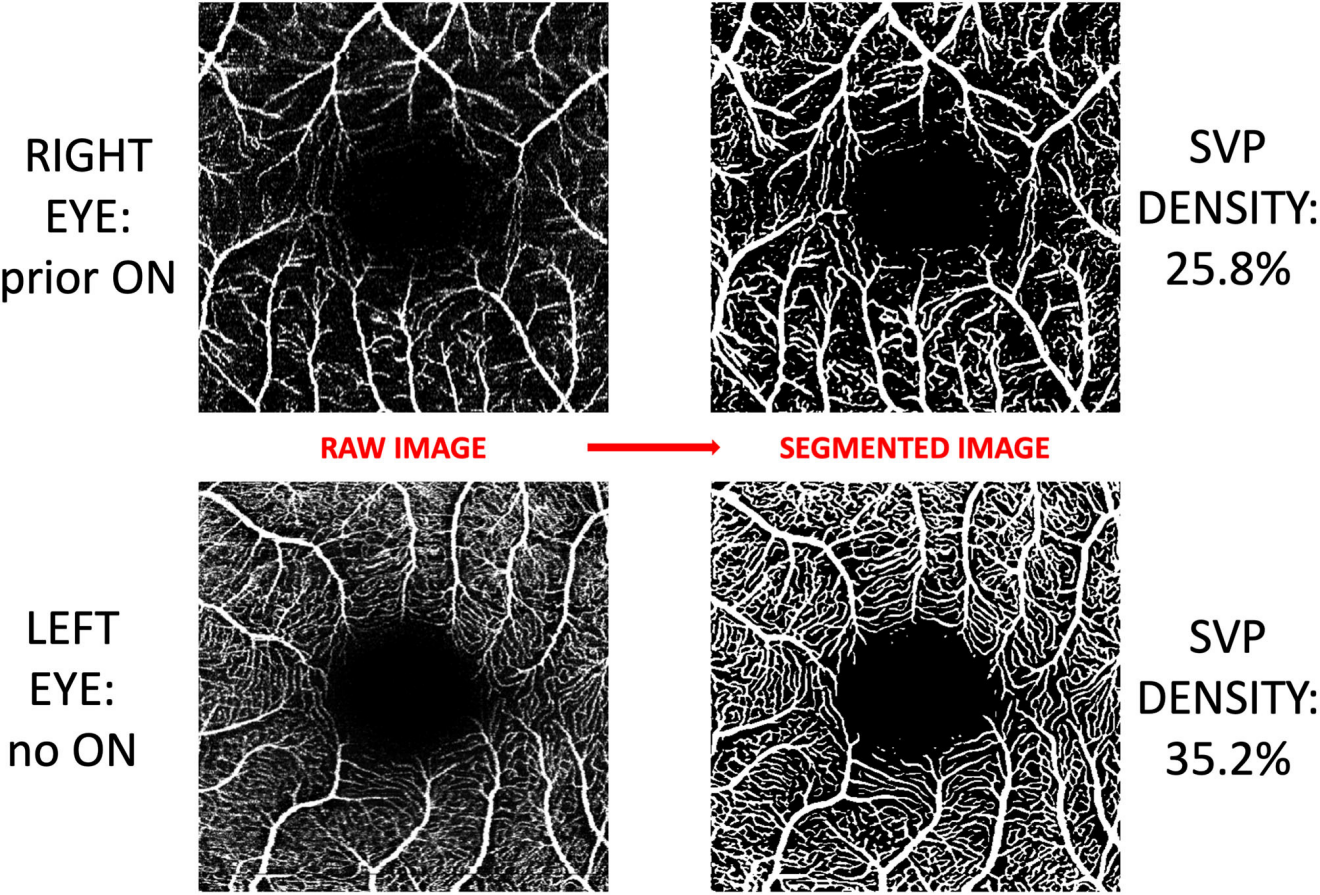
Post-acquisition processing and segmentation of OCTA images. Images of the superficial vascular plexus (SVP) were acquired using Spectralis OCTA (Heidelberg Engineering, Germany). A neural network algorithm developed at Johns Hopkins University was trained to exploit the shared vessel structures from repeated scans acquired by two different OCTA devices while suppressing the scan-dependent noises and artifacts. The trained algorithm generates a vessel mask for each scan for computing SVP density. In this figure, OCTA images from both eyes of a patient with a history of optic neuritis are demonstrated. Inter-eye difference in SVP density in this patient was −9.4%.

Macular OCTA scans were acquired using Spectralis SD-OCTA (model Spec-CAM S2610 with OCT2 and OCTA modules, software version 6.9a-US-IRB, Heidelberg Engineering, Germany), with TruTrack active eye tracking and full-spectrum amplitude decorrelation algorithm (FS-ADA) for motion detection and image generation. The device acquires 85,000 A-scans per second at an axial resolution of 7 um, using a wavelength of 870 nm. We excluded images with a sustained signal strength of <25 dB. 3 × 3 mm images were automatically segmented by the device into the SVP and deep vascular plexus (DVP). Density of the SVP was quantified using a deep neural network which reduces imaging artifact, improves signal-to-noise ratio of OCTA images, and increases accuracy of derived SVP density measurements. This neural network algorithm was developed at Johns Hopkins University using a variational intensity cross channel encoder that finds vessel masks by examining the common vascular architecture shared by OCTA images of the same region acquired using different OCTA devices (Figure 1) (17). Rigorous quality control criteria were applied to the acquisition and processing of OCTA images consistent with prior publications by our group (6). OCTA imaging artifact was graded by a single rater (OCM), by viewing each raw OCTA image side-by-side with the segmented image which represents an artifact-reduced vessel mask. Segmented images which demonstrated impaired detection of >25% of the capillary micro-architecture were excluded from analyses (Table 1).

**Table 1:** OCTA image quality control.

	**All patients** **(*n* = 63)**	**MS ON** **patients**	**MS Non-ON** **patients**
		**(*n* = 45)**	**(*n* = 18)**
Total OCTA images, *n* eyes	96	224	72
Minor image artifact (<25% of the capillary architecture), *n* eyes	260 (88%)	202 (90%)	58 (81%)
Major image artifact (>25% of the capillary architecture), *n* eyes	36 (12%)	22 (10%)	14 (19%)

*Each raw OCTA image was viewed side-by-side with the post-processing artifact-reduced segmented image, and the proportion of the segmented image demonstrating impaired detection of the capillary micro-architecture was classified as <25 or >25%. Images with major artifact were excluded from all further analyses*.

### Visual Function Assessment

Visual function was measured monocularly and patients were instructed to use their habitual spectacles or contact lens where applicable. Retro-illuminated Early Treatment of Diabetic Retinopathy study charts were utilized at 4 m to assess 100% letter acuity (LA), while low-contrast Sloan letter charts were utilized at 2 meters to assess 2.5% and 1.25% LA. LA scores were calculated based on the number of correct letters achieved in each chart.

### Calculation of Inter-Eye Differences

IEDs in OCT, OCTA, and visual function measures were calculated with respect to the ON eye in MS ON patients, and with respect to the right eye in MS non-ON patients. For example, in a patient with a history of optic neuritis in the right eye, and a 100% contrast LA score of 65 in the right eye and 68 in the left eye, the IED for this measure would be −3.

### Statistical Analyses

MS ON patients were compared to MS non-ON patients in initial analyses of baseline demographics and IEDs in OCT/OCTA measures and LA. MS ON patients were also grouped into 4 categories for further analyses, according to the time that had elapsed between the onset of ON, and the acquisition of OCT/OCTA images (<3 months, 3 to 12 months, 1–3 years, >3 years). Statistical analyses were completed using Stata version 16 (StataCorp, College Station, TX, United States). Wilcoxon rank sum test or the chi-squared test were used to compare demographic variables between the MS ON group and the MS non-ON group. For all other analyses, we used mixed-effects linear regression analyses accounting for intra-subject correlations. *R*^2^-values were estimated using the Snijders and Bosker method (18). We used unadjusted models, since variables which may affect OCT/OCTA measurements in individual eyes (e.g., age, sex, race, disease duration) are not thought to have the same degree of confounding effect on IEDs. *P*-value for significance was defined as <0.05.

### Ethical Approval

Johns Hopkins University institutional review board approval was obtained for study protocols, and all patients provided written informed consent.

## Results

### Demographic and Clinical Characteristics

All SVP imaging sets underwent quality control protocols and image artifact rating (Table 1). After exclusion of imaging sets in which one or both eyes were affected by major artifact, our study population comprised 43 MS ON patients (with 92 study visits) and 14 MS non-ON patients (with 24 study visits). On average, MS ON patients were younger and had a shorter MS disease duration than MS non-ON patients (*p* = 0.001 for both, Table 2).

**Table 2:** Demographic and clinical characteristics of participants.

	**MS ON**	**MS Non-ON**	***P*-value**
	**patients**	**patients**	
*N*, participants	43	14	–
*N*, visits	92 (184 eyes)	24 (48 eyes)	–
Age, mean years (SD)	33.7 (9.3)	44.4 (7.7)	0.001^a^
Female, *n*	36 (84%)	11 (78%)	0.66^b^
**Race**			
Caucasian, *n*	29 (67%)	12 (86%)	0.18^b^
African/African-American, *n*	13 (33%)	1 (7%)	
Other, *n*	1 (2%)	1 (7%)	
Disease duration, mean years (SD)	4.5 (6.2)	14.9 (4.8)	<0.001^a^

Imaging sets in which one or both SVP images were affected by major artifact were excluded. ON, optic neuritis; SD, standard deviation.

a Wilcoxon rank sum test,

b *Chi-squared test*.

### Inter-Eye Differences in OCT, OCTA, and Visual Function Measures

**Figure 2:**
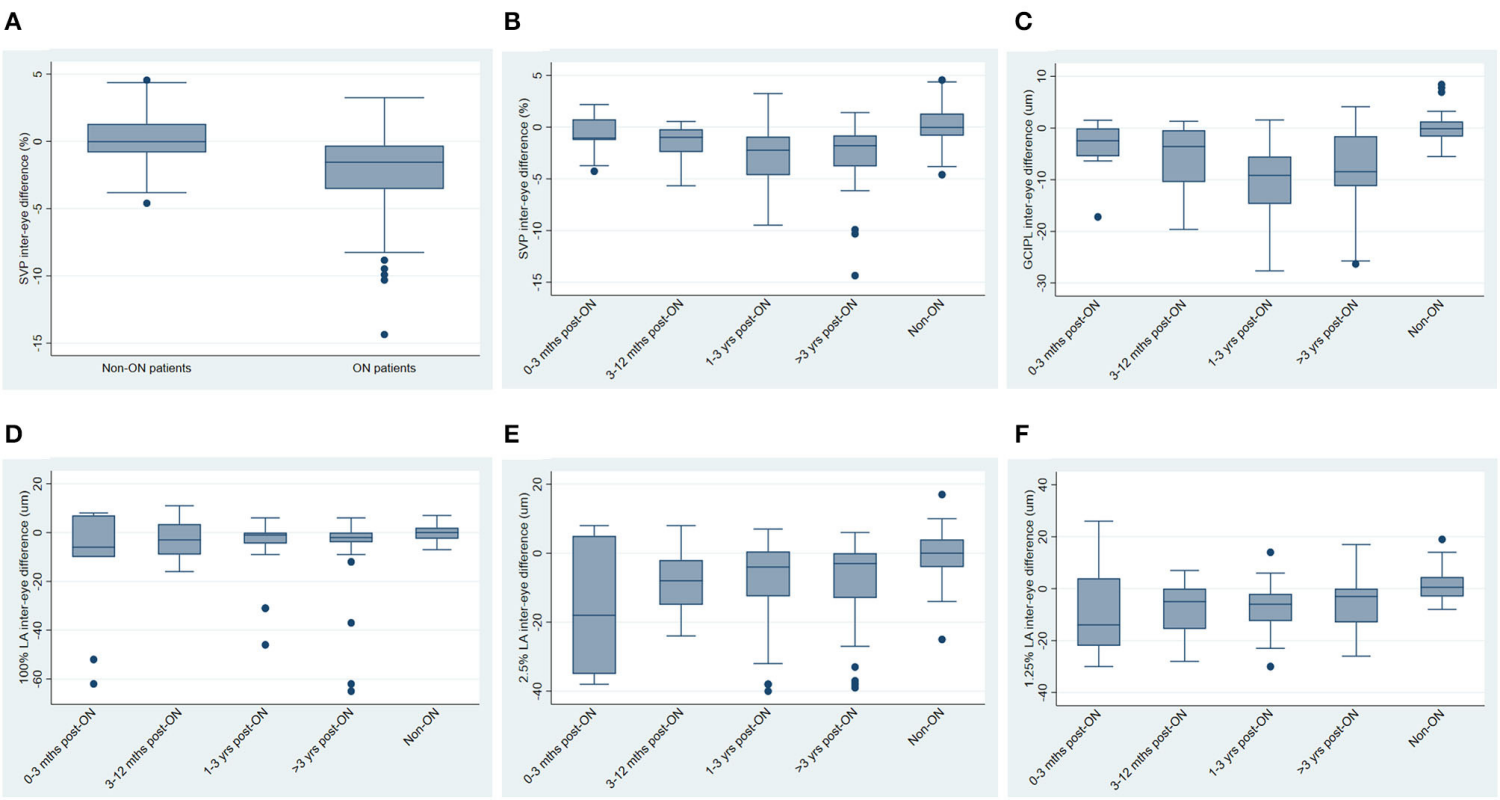
Inter-eye differences in SVP density, GCIPL thickness and visual function, stratified by history of optic neuritis**. (A)** Inter-eye differences in SVP density in MS non-ON patients vs. MS ON patients. **(B)** Inter-eye differences in SVP density in MS ON patients stratified by time from ON, vs. MS non-ON patients. **(C)** Inter-eye differences in GCIPL thickness in MS ON patients stratified by time from ON, vs. MS non-ON patients. **(D–F)** Inter-eye differences in 100% contrast letter acuity **(D)**, 2.5% contrast letter acuity **(E)**, and 1.25% contrast letter acuity **(F)** in MS ON patients stratified by time from ON vs. MS non-ON patients.

IEDs in SVP density were larger in MS ON patients as compared to MS non-ON patients [mean −2.69% (SD 3.23) vs. mean 0.17% (SD 2.39) *p*=0.002]. Compared to MS non-ON patients, MS ON patients also demonstrated significantly larger IEDs in pRNFL thickness, GCIPL thickness, AMT, 2.5% contrast LA, and 1.25% contrast LA (Table 3). MS ON patients were then stratified into groups according to time from ON and compared to the reference group of MS non-ON patients in multiple cross-sectional analyses (Table 4, Figure 2). Compared to the MS non-ON group, IEDs in GCIPL thickness, pRNFL thickness, and AMT were significantly larger in MS ON patients at 3–12 months post-ON, 1–3 years post-ON, and >3 years post-ON. Additionally, relative to non-ON MS patients, larger IEDs in GCIPL thickness were even detectable in MS ON patients <3 months post-ON (*p* = 0.01). Regarding IEDs in SVP density, larger differences were identified in MS ON patients at 1–3 years and >3 years post-ON, as compared to MS non-ON patients, whereas differences in the earlier timeframes (<3 months and 3–12 months) were not significant. It is worth noting that the MS ON groups at 1–3 years and >3 years post-ON showed a greater variation in IEDs of GCIPL thickness, pRFNL thickness, AMT, and SVP density (Figure 2), which may impact the interpretation and comparison of the multiple cross-sectional analyses illustrated in Table 4.

**Table 3:** Inter-eye differences in OCT, OCTA, and visual function measures in MS ON vs. MS non-ON patients.

	**MS ON**	**MS Non-ON**	***P*-value**
	**patients**	**patients**	
*N*, participants	43	14	–
*N*, visits	92 (184 eyes)	24 (48 eyes)	–
pRNFL IED, mean % (SD)	−9.49 (15.05)	2.29 (6.21)	0.001
GCIPL IED, mean % (SD)	−9.34 (8.48)	0.87 (3.63)	<0.001
INL IED, mean % (SD)	0.11 (1.76)	0.35 (0.97)	0.82
ONL IED, mean % (SD)	0.29 (1.52)	0.48 (1.30)	0.46
AMT IED mean % (SD)	−13.05 (12.0)	2.97 (6.63)	<0.001
SVP IED, mean % (SD)	−2.69 (3.23)	0.17 (2.39)	0.002
100% contrast LA IED, mean (SD)	−7.3 (15.8)	−0.06 (3.3)	0.06
2.5% contrast LA IED, mean (SD)	−11.5 (14.1)	−0.1 (8.2)	0.004
1.25% contrast LA IED, mean (SD)	−8.2 (12.0)	1.9 (6.6)	0.002

*IED, inter-eye difference (using the ON eye as the reference eye in MS ON patients, and using the right eye as the reference eye in MS non-ON patients). ON, optic neuritis; SD, standard deviation; pRNFL, peripapillary retinal nerve fiber layer thickness; GCIPL, ganglion cell + inner plexiform layer thickness; INL, inner nuclear layer thickness; ONL, outer nuclear layer thickness; AMT, average macular thickness; LA, letter acuity. P-values were calculated using mixed effects linear regression accounting for intra-subject correlations*.

**Table 4:** Inter-eye differences in OCT, OCTA, and visual function measures, with MS ON patients stratified by time from ON.

	**MS ON: <3** **months post-ON**	**MS ON: 3–12** **months post-ON**	**MS ON: 1–3** **years post-ON**	**MS ON: >3** **years post-ON**	**MS Non-ON**	***P*-value (<3 months** **vs. non-ON)**	***P*-value (3-12 months** **vs. non-ON)**	***P*-value (1-3 yrs** **vs. non-ON)**	***P*-value (>3 yrs** **vs. non-ON)**
N	13 visits in 9 patients	12 visits in 12 patients	37 visits in 18 patients	30 visits in 19 patients	24 visits in 14 patients	–	–	–	–
pRNFL IED, mean um (SD)	10.25 (18.63)	−6.17 (7.66)	−15.69 (11.68)	−14.02 (12.83)	2.29 (6.21)	0.15	**0.001**	**<0.001**	**<0.001**
GCIPL IED, mean um (SD)	−4.99 (6.34)	−5.74 (6.98)	−11.21 (8.59)	−11.22 (9.11)	0.87 (3.63)	**0.01**	**0.001**	**<0.001**	**<0.001**
INL IED, mean um (SD)	0.14 (0.67)	−0.32 (1.03)	−0.08 (1.35)	0.54 (2.53)	0.35 (0.97)	0.65	0.13	0.30	0.80
ONL IED, mean um (SD)	2.25 (2.19)	1.12 (1.31)	−0.09 (0.90)	−0.50 (1.12)	0.48 (1.30)	**0.04**	0.20	0.15	**0.02**
AMT IED, mean um (SD)	−3.54 (6.76)	−7.13 (7.66)	−16.85 (12.95)	−16.21 (12.11)	2.97 (6.64)	0.05	**<0.001**	**<0.001**	**<0.001**
SVP IED, mean % (SD)	−1.09 (2.14)	−1.45 (1.75)	−3.17 (3.20)	−3.79 (3.96)	0.17 (2.39)	0.21	0.07	**0.001**	**0.001**
100% contrast LA IED, mean (SD)	−12 (16)	−2 (8)	−4 (10)	−7 (17)	0 (3)	0.02	0.22	0.09	0.06
2.5% contrast LA IED, mean (SD)	−15 (15)	−8 (10)	−8 (12)	−9 (14)	0 (7)	**0.002**	**0.01**	**0.02**	**0.007**
1.25% contrast LA IED, mean (SD)	−11 (17)	−7 (11)	−8 (8)	−6 (10)	2 (6)	**0.04**	**0.004**	**0.001**	**0.004**

*IED, inter-eye difference (using the ON eye as the reference eye in MS ON patients, and using the right eye as the reference eye in MS non-ON patients). ON, optic neuritis; pRNFL, peripapillary retinal nerve fiber layer thickness; GCIPL, ganglion cell + inner plexiform layer thickness; INL, inner nuclear layer thickness; ONL, outer nuclear layer thickness; AMT, average macular thickness; LA, letter acuity. Mean IEDs indicated were calculated using average values per subject within each timeframe. P-values were calculated using mixed effects linear regression accounting for intra-subject correlations. Bold indicates p value for significance of < 0.05*.

### Relationships Between OCT, OCTA, and Visual Function Measures

**Figure 3:**
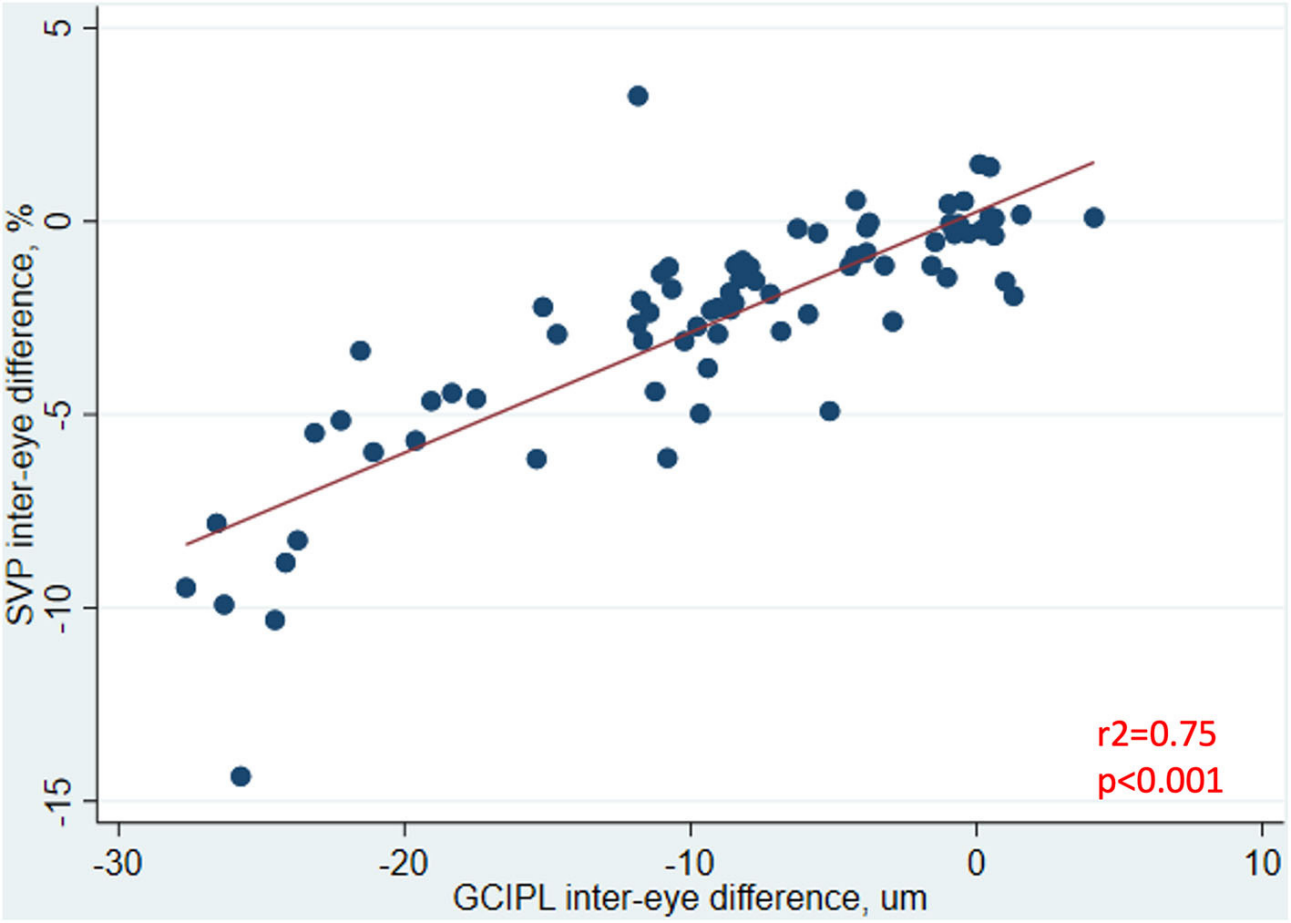
Relationship between inter-eye differences in SVP density and GCIPL thickness in MS ON patients. In MS ON patients at least 3 months post-ON (after resolution of the acute phase of ON), the relationship between inter-eye differences in SVP density and GCIPL thickness is shown here. *P*-value was calculated using mixed-effects linear regression accounting for intra-subject correlations. *R*^2^-value was estimated from the mixed-effects model using the Snijders and Bosker method.

**Figure 4:**
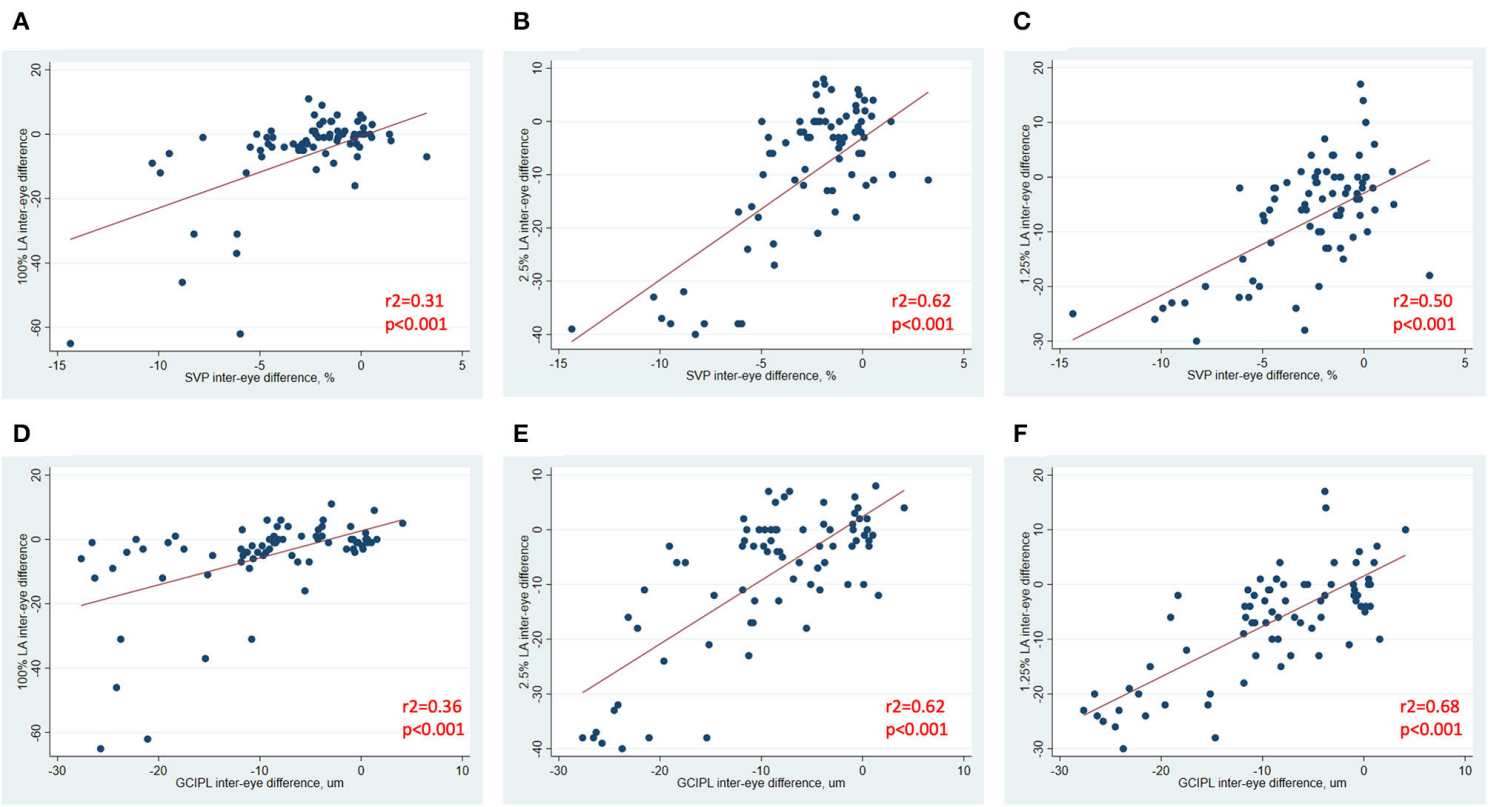
Relationships between inter-eye differences in OCT/OCTA and visual function measures in MS ON patients. In MS ON patients at least 3 months post-ON (after resolution of the acute phase of ON), relationships between inter-eye differences in SVP density and 100% contrast LA, 2.5% contrast LA, and 1.25% contrast LA are demonstrated in **(A–C)**. In MS ON patients at least 3 months post-ON, relationships between inter-eye differences in GCIPL thickness and 100% contrast LA, 2.5% contrast LA, and 1.25% contrast LA are demonstrated in **(D–F)**. *P*-values were calculated using mixed-effects linear regression accounting for intra-subject correlations. *R*^2^-values were estimated from the mixed-effects model using the Snijders and Bosker method.

**Figure 5:**
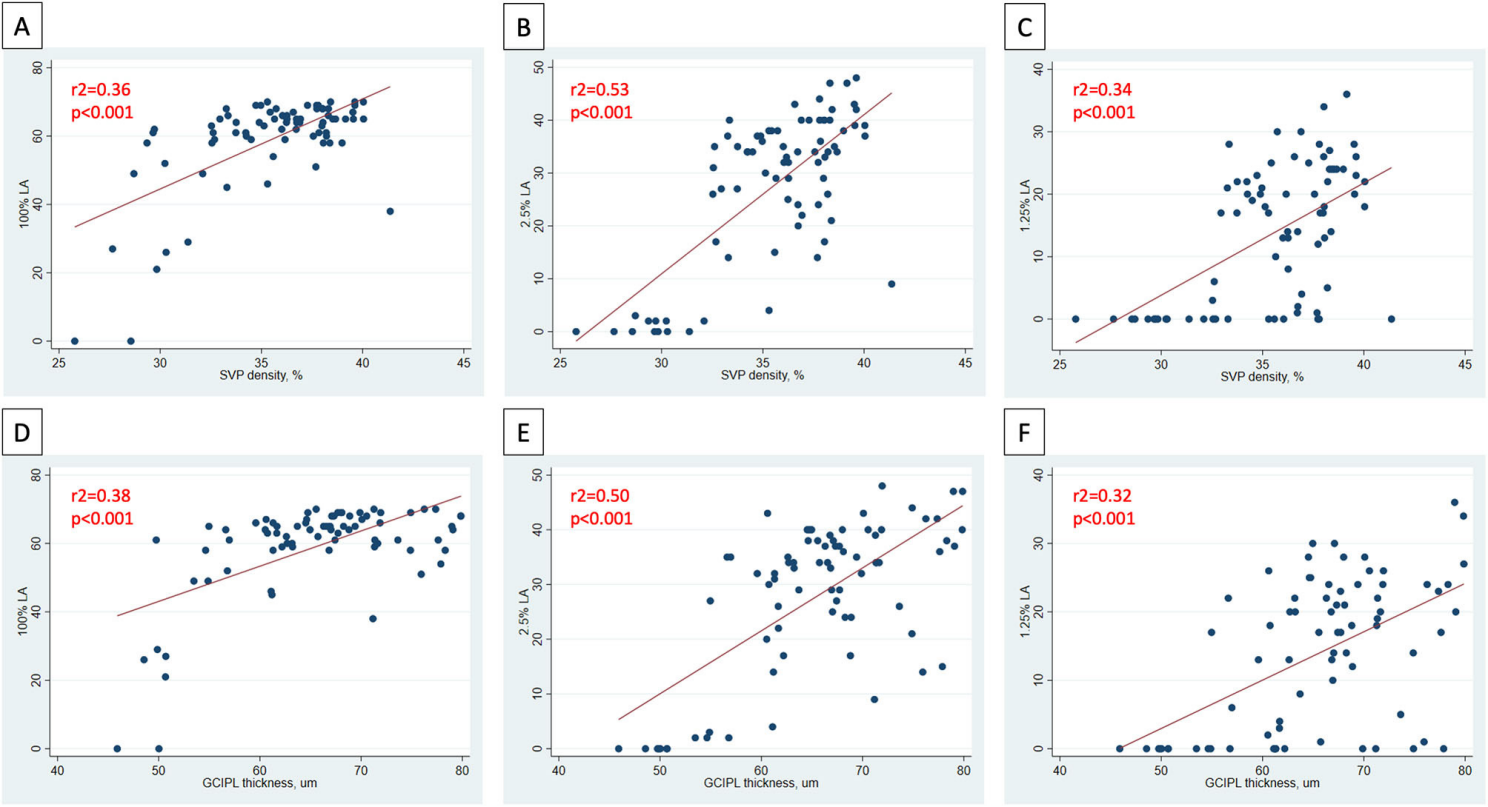
Relationships between OCT/OCTA and visual function measures in individual MS ON eyes. In MS ON eyes at least 3 months post-ON (after resolution of the acute phase of ON), relationships between SVP density and 100% contrast LA, 2.5% contrast LA, and 1.25% contrast LA in the MS ON eye are demonstrated in **(A–C)**. In MS ON eyes at least 3 months post-ON, relationships between GCIPL thickness and 100% contrast LA, 2.5% contrast LA, and 1.25% contrast LA in the MS ON eye are demonstrated in **(D–F)**. *P*-values were calculated using mixed-effects linear regression accounting for intra-subject correlations. *R*^2^-values were estimated from the mixed-effects model using the Snijders and Bosker method.

In analyses of MS ON patients excluding visits during the acute phase (i.e., excluding visits <3 months post-ON), a strong relationship was identified between larger IEDs in SVP density and larger IEDs in GCIPL thickness (*r*^2^ = 0.75, *p* < 0.001, Figure 3), and between larger IEDs in SVP density and larger differences in 100% contrast LA (*r*^2^ = 0.31, *p* < 0.001), 2.5% contrast LA (*r*^2^ = 0.62, *p* < 0.001), and 1.25% contrast LA (*r*^2^ = 0.50, *p* < 0.001, Figure 4). In the same patients, significant relationships were also identified between IEDs in GCIPL thickness and IEDs in 100% contrast LA (*r*^2^ = 0.36, *p* < 0.001), 2.5% contrast LA (*r*^2^ = 0.62, *p* < 0.001), and 1.25% contrast LA (*r*^2^ = 0.68, *p* < 0.001, Figure 4). We also examined whether relationships between OCT/OCTA and visual function measures in individual MS ON eyes may differ to the relationships seen between inter-eye differences in the same measures. Results using measures from individual MS ON eyes were broadly similar to those identified using inter-eye differences (Figure 5).

## Discussion

Results of our study show that IEDs in SVP density are larger in MS ON patients than in MS non-ON patients. Additionally, we found significant relationships between IEDs in SVP density and visual function measures in MS ON patients (after the acute phase of ON), and similar relationships were also identified between IEDs in GCIPL thickness and visual function. ON eyes that exhibit greater reductions of SVP density or GCIPL thickness relative to the fellow eye are more likely to have greater reductions of high- and low-contrast LA scores. We also identified potential differences between the temporal dynamics of OCT and OCTA measures after ON, as increased IED in GCIPL thickness was detectable within 3 months after ON onset, while increased IED in SVP density was only detectable at least 1 year after ON. GCIPL thickness is already considered an excellent surrogate of neuroaxonal injury after ON due to its reliability, reproducibility, and strong structure-function relationships, and our data suggest that SVP density may offer additional insights into pathobiological processes and have value as a biomarker of visual outcomes following ON.

Multiple studies have demonstrated reduced vascular plexus densities or flow indices in patients with MS, both at the optic nerve head and macula (6–9, 19–22). In the macula, SVP density has been consistently shown to be reduced in patients with MS compared to healthy controls, with reductions being greatest in eyes with a history of ON (6–9, 22). Furthermore, relationships between reduced SVP density and poorer visual function or higher global disability measures have been shown in a number of studies (6, 8). Some of the relationships between SVP density and disability measures have been stronger than those identified between GCIPL thickness and disability measures in the same patients (6). The reason for this is uncertain, but we have hypothesized that reductions SVP density may to some extent reflect not only reduced retinal tissue volume, but perhaps also provide insights into the metabolic function (or indeed dysfunction) of surviving tissue. Due to the relationships with disability measures, and good reliability and reproducibility (23, 24), OCTA has emerged in recent years as an attractive biomarker in MS. However, OCTA has a number of limitations in this population which may hamper interpretation of findings in individual patients, including quite a high frequency of imaging artifact (25) and lack of accepted normal ranges for vascular plexus densities that can be applied across devices in healthy individuals. Thus, the potential clinical utility of a single OCTA evaluation in patients with MS has not been well-demonstrated to date. To this end, examining IEDs can provide clinically-useful information at a single timepoint for individual patients, and we have demonstrated the important finding that IED in SVP density is increased after ON in MS.

The dynamics of changes in retinal layer thicknesses after ON have been well-described (26–28). The acute phase of ON is typically characterized by increased pRNFL thickness in ON eyes due to the effects of acute inflammation within the optic nerve (26, 27), and increased INL thickness may also be detectable during this phase (27). Edema and other components of the acute inflammatory process resolve over the subsequent 3–4 months, and the pRNFL and GCIPL layers undergo rapid thinning during this period reflecting neuroaxonal loss (27). By around 4–6 months after onset of ON, a new baseline in the ON eye appears to be established, and rates of retinal layer atrophy revert to being comparable to the fellow (non-ON) eye (27, 29). Over the long-term, the rate of change of GCIPL thickness may actually be lower in ON eyes than in non-ON eyes (since the proportion of tissue lost annually may be smaller when the baseline thickness is lower) (30). On the other hand, the temporal dynamics of OCTA changes following ON are not well-understood. Our data suggest that increased IED in SVP density is clearly established by 1 year after ON, but these differences may not be detectable within the initial 12 months after ON. While retinal tissue loss is likely to be a primary driver of reduced SVP density following ON, the reason for the potential temporal disconnect between these detectable changes is uncertain, and there are a number of potential explanations. First, post-ON changes in the retinal tissue structure and retinal vasculature may not be synchronous, and we hypothesize that reductions in SVP density may not only reflect initial tissue loss incited by inflammation-related axonal injury in the optic nerve, but may also be a marker of later reduced metabolic demand in injured but surviving tissue. Second, we noted that the severity of the retinal tissue loss appeared greater in our group of patients who were >1 year post-ON, and perhaps significant IEDs in SVP density are only detectable in cases where there is greater tissue injury. Third, the number of patients in our cohort who were <12 months post-ON was smaller than the number of patients who were >1 year post-ON, and so we may have been underpowered to detect differences within the earlier timeframes. Additionally, while SVP density and GCIPL thickness are known to be closely related cross-sectionally, it is not yet understood whether changes in these measures occur *proportionately* following ON–representing an important question for future longitudinal studies.

IEDs in retinal layer thicknesses are accepted as an informative and clinically-applicable way to measure retinal changes after optic neuritis. Since retinal layer thicknesses exhibit high inter-individual variability (even in healthy subjects), ON eyes may experience substantial tissue loss without the actual pRNFL or GCIPL thickness dropping into the “abnormal” range (considered <5th percentile in clinical practice) (31). This may partially explain why retinal layer thicknesses in individual eyes correlate well with history of ON and monocular visual function at a group level (32, 33), but monocular eye thresholds may have limited value for defining ON in individual patients (31, 34). IEDs in retinal layer thicknesses have been proposed as a more informative marker of ON in individuals, since the reliability and reproducibility of OCT-derived retinal layer thicknesses (particularly GCIPL thickness) is high, and the symmetry between right and left eyes in healthy individuals is also high (31, 34–36). IED in GCIPL thickness has emerged as a reasonably robust marker of prior ON, with a threshold of >4 um demonstrating reasonably good sensitivity and specificity for discriminating ON history (34, 35). Additionally, IED in GCIPL thickness has shown significant associations with visual function outcomes after ON (34). In our study, IED in SVP density showed similar correlations with low contrast LA (recognized as a more sensitive marker of visual dysfunction than high contrast VA in MS) (37, 38) as those identified with GCIPL thickness. This is in keeping with findings of prior work by our group, in which both SVP density and GCIPL thickness were found to correlate with EDSS and LA scores in MS patients, and SVP density was additionally found to correlate with MSFC scores (6). OCTA images are generated through the detection of motion within blood vessels according to a threshold effect, so reduced SVP density may reflect reduced tissue volume as well as reduced blood flow within surviving tissue due to tissue dysfunction or hypometabolism. For these reasons, SVP density may offer additional insights into tissue function, that is perhaps reflected in the associations we identified with high- and low-contrast LA. Another potential advantage that OCTA may offer in post-ON eyes, is that the derived vascular measures might not be affected by the same “floor” phenomenon as OCT measures (whereby retinal tissue loss may be undetectable below a certain threshold) (39).

In considering the potential importance of changes in OCTA findings following ON, it is essential to remember that reductions in macular and peripapillary vascular measures have also been reported in other etiologies of optic neuropathy such as glaucoma and non-arteritic anterior ischemic optic neuropathy (NAION) (40–42). NAION eyes demonstrate similar although usually more marked reductions in SVP density to ON eyes (40), while glaucomatous optic neuropathy may be associated with changes in deep vascular plexus (DVP) density as well as SVP density (41). While all of these optic neuropathies are associated with loss of retinal ganglion cells, the different patterns of retinal vascular changes suggests that OCTA may provide clues regarding underlying pathophysiology, requiring further exploration in future studies.

Our study is novel in its approach to examine IEDs in both OCT and OCTA measures after ON in MS. We have employed careful quality control protocols and an effective neural network based-approach to systematically reduce the impact of artifact on OCTA analyses. However, our study has a number of limitations. Our study population was relatively small, and we may have been underpowered to detect differences in OCTA measures in all of the MS ON subgroups. The MS non-ON patients were on average older, had a longer MS disease duration, and were more frequently Caucasian than the MS ON patients, and it is uncertain whether age, disease duration, or race alone may have a substantial impact on the symmetry of OCTA measures between eyes in individuals. The differences in OCTA measures between African-American and Caucasian-American people with MS represents an important question for future research in larger cohorts, since it is known that African-American people with MS tend to have worse visual outcomes after ON and accelerated rates of retinal atrophy, compared to Caucasian-American people with MS (43–45). Our multiple cross-sectional comparisons included different patients at different timepoints after ON, and some patients may have experienced a greater severity of acute ON than others. Since OCTA is a relatively new technology and we have not been tracking patients for many years with this technique, we were unable to perform a longitudinal analysis to get a robust picture of the temporal dynamics of OCTA changes after ON. This represents a key area for future research. Another important area of further study would include longitudinal evaluation of not just SVP density, but also DVP density and whole vessel density in ON eyes.

## Conclusions

Increased IED in SVP density in MS patients after ON can be detected using OCTA, and detectable changes in SVP density after ON may occur slightly later than those changes in GCIPL thickness. Additionally, IED in SVP density demonstrates robust correlations with visual function in MS ON patients. Our findings support the potential clinical utility of OCTA for detecting ON-related changes in patients with MS. Furthermore, our results provide important insights into the interplay between retinal tissue changes and retinal vascular changes following ON. OCTA represents a rapid reliable technique that may provide additional clinically-relevant information beyond standard OCT techniques in MS patients, and represents a potential biomarker of post-ON outcomes for future clinical trials.

## Data Availability Statement

The raw data supporting the conclusions of this article will be made available by the authors, subject to institutional review board approval and without undue reservation.

## Ethics Statement

The studies involving human participants were reviewed and approved by Johns Hopkins University Institutional Review Board. The patients/participants provided their written informed consent to participate in this study.

## Author Contributions

OM was involved in study conceptualization and planning, data acquisition and analysis, data interpretation, drafting, and revising the manuscript. GK, EV, AF, JL, HE, NP, ES, NL, and YL were involved in data acquisition and analysis, data interpretation, and revising the manuscript. KF, JP, and PC were involved in data interpretation, and revising the manuscript. SS was involved in study conceptualization and planning, data acquisition and analysis, data interpretation, and revising the manuscript. All authors contributed to the article and approved the submitted version.

## Conflict of Interest

ES has served on a scientific advisory boards for Viela Bio and Genentech and is funded by a Sylvia Lawry physician fellowship award from NMSS. JP is a founder of Sonovex, Inc. and serves on its Board of Directors. He has received consulting fees from JuneBrain LLC and is PI on research grants to Johns Hopkins from 12Sigma Technologies and Biogen. PC has received consulting fees from Disarm and Biogen and is PI on grants to JHU from Biogen and Annexon. SS has received consulting fees from Medical Logix for the development of CME programs in neurology, and has served on scientific advisory boards for Biogen, Genzyme, Genentech Corporation, EMD Serono, and Celgene. He is the PI of investigator-initiated studies funded by Genentech and Biogen, was the site investigator of a trial sponsored by MedDay Pharmaceuticals, and received support from the Race to Erase MS foundation. He has received equity compensation for consulting from JuneBrain LLC, a retinal imaging device developer. The remaining authors declare that the research was conducted in the absence of any commercial or financial relationships that could be construed as a potential conflict of interest.
